# The Pain Research Enrichment Program (PREP): Developing an immersive program for research assistants at an academic medical center

**DOI:** 10.1017/cts.2024.489

**Published:** 2024-02-26

**Authors:** Jennifer Pierce, Sana Shaikh, Caroline S. Zubieta, Guohao Zhu

**Affiliations:** Department of Anesthesiology, University of Michigan, Ann Arbor, MI, USA

**Keywords:** Job satisfaction, staff training, staff morale, staff well-being, temporary research assistants

## Abstract

Research experience is often important for academic and career development. This paper describes the implementation and impact of a training program for temporary research assistants (RAs) at an academic medical center. The program includes a 9-month didactic lecture series covering research and professional development skills, a Quality Improvement project focused on improving research processes, and manuscript writing. Overall, the program goals of increasing confidence, self-efficacy, job satisfaction, and well-being, as well as providing an opportunity for career exploration, were met. Thus, this program has the potential to support temporary RAs and enhance their early research experiences.

This paper describes the development and early implementation of a training program, the Pain Research Enrichment Program (PREP), for temporary research assistants (RAs, i.e., undergraduate, gap year, and medical students) in the Division of Pain Research, Department of Anesthesiology at the University of Michigan. The goal of the program is to offer opportunities to develop research and professional development skills and to provide an enhanced research experience beyond job experience alone.

Temporary RAs are often college students or recently graduated, which is a period marked by stress and academic- and career-related anxiety that may have been exacerbated due to the COVID-19 pandemic [1,2,7]. This stress may be mitigated by supportive training prior to entering the workforce. Indeed, previous research supports the benefit of active learning behaviors and practical skill building on sense of preparedness for future work among college graduates [4]. Many young adults seek employment with research teams to gain experience that will aid them in future career endeavors. For example, admissions into graduate and medical school programs, as well as future medical internships and residencies, are intensely competitive and research experience is often considered a boon to the application that can springboard individuals into the select few who are considered for acceptance. These transitory research positions are prime opportunities to enhance research and professional development skills to provide a firm foundation early in individuals’ careers, as well as to provide support for career exploration.

Despite the apparent need for training and support, many RAs have lackluster research experiences. Many early research experiences involve the drudgery of research, such as data entry, participant recruitment, and benchwork, rather than study conceptualization, implementation, and manuscript writing. Although the former work is critically important to the success of any team, the latter experiences can offer RAs more holistic experiences to determine whether they have a passion for research. These experiences may also improve RAs’ sense of self-efficacy, investment, perceived control over their work, and motivation, thereby enhancing well-being [3]. Importantly, it is also possible to harness the research-related work experiences of RAs who are embedded in the process of recruitment, study visits, participant communication, and retention to enhance research processes. Additionally, by pursuing research questions that are relevant to the important work they are engaged in as opposed to a traditional research project, this may further magnify the sense of importance in the work that RAs do as well as improve RAs’ understanding of the operational aspects of clinical and translational research.

The Back & Pain Center (B&PC) at Michigan Medicine is a tertiary-care, outpatient pain clinic that houses a robust research team. The research team at the B&PC is part of the Division of Pain Research, which encompasses additional research teams across Michigan Medicine. The research teams in the Division of Pain Research include several permanent members, with the team at the B&PC in particular including research coordinators, a data manager, a statistician (GZ), and a program manager (SS). However, many RA positions are temporary, filled by transient undergraduate, gap year, and graduate or medical students. JP and SS sought to develop a training program that would enhance their research training to build confidence and self-efficacy, improve job satisfaction and well-being, as well as provide experiences that supported career exploration.

Our pilot program, first launched by JP and SS in fall 2019, included monthly lectures covering numerous topics, such as developing a research question, basics of statistics, poster presentations, networking, and interview skills. There was also a final poster project utilizing existing data. The participants formed teams to develop a research question and hypothesis and then worked with JP and SS through data analysis and poster creation. Yet, midway through the pilot program, COVID-19 began, and all conferences were canceled. Because the research poster was considered an important aspect of the training goals and the conference landscape abruptly changed, JP and SS sought an alternative. We determined that we could maximize career exploration and skill building, while avoiding the limitations of conference cancelations, by focusing on manuscript writing; however, the secondary data sources that we had available would lead to disparate research questions or may not fit RAs' interests. A Quality Improvement (QI) project that focused on the practical skills they were building as RAs, exposed them to important operational aspects of research that could carry forward to a career in research, and that could also potentially improve our processes would be ideal. Therefore, in summer 2020, JP and SS recruited a small group of RAs to develop a QI project which culminated in a low-effort online survey project that was completed in concordance with the didactic series. The result was a highly engaging research experience that directly related to RAs’ ongoing research activities (i.e., concerns regarding participant recruitment and retention) with deliverables (i.e., manuscripts and posters).

## Program structure

Temporary RAs are in the Division of Pain Research who are interested in participating in the program contact JP or SS; as of now, participation is not competitive and all those interested are invited to participate. The revised PREP program encompasses three primary components: didactic lectures, a QI project, and manuscript writing. Each component is described below.

## Didactic lectures

Lectures are provided roughly every 2 weeks across 9 months with breaks centered around the academic calendar. This results in roughly 12 lectures per cohort. The lectures are primarily provided by guests who are experts in that topic. Lectures last for approximately 1 hour, can be attended remotely, and are held during work hours with support from participants’ supervisors. Recorded lectures are posted in a shared online folder. See Table 1 for a sample schedule.

**Table 1.** Sample PREP lectures

Week	Lecture topic
1	Introduction and program goals
2	QI project brainstorming session
3	How to do a literature search
4	How to “mentor up”
5	Basics of statistics
6	Interviewing skills for medical/graduate school and residency
7	How to develop a research question
8	Valid informed consent and ethics of data collection
9	QI project check-in
10	DEI in research
11	Interpersonal skills in research
12	How to write a manuscript
13	Presentation skills
14	Grant writing basics
15	Institutional Review Board application basics
16	Program wrap-up

PREP = Pain Research Enrichment Program. QI = Quality Improvement. DEI = Diversity, Equity, and Inclusion.

These are lecture topics currently being offered to the 2023–2024 cohort. Lecture topics have varied over the cohorts as the program has grown and participant feedback has been obtained.

## QI project

A QI project is developed focused on improving research processes at the B&PC. The QI project is intended to be low effort and, therefore, involve an online, cross-sectional survey with low burden recruitment and participant tracking. Undergraduate RAs from the University of Michigan’s Undergraduate Research Opportunity Program (UROP), which is a university-wide program that supports the involvement of undergraduate students in research projects across an academic year, are recruited to join as temporary RAs at no cost to the B&PC to assist with the QI project. UROP students are then also participants in the PREP program. UROP students who have participated in PREP have typically worked about 6 hours per week. PREP participants work with the program directors (JP, SS, CSZ, and GZ) to develop hypotheses, identify appropriate survey measures, construct a codebook, build the online survey, and write the Institutional Review Board application. These research questions come together to form a single QI project.

## Manuscript writing

PREP participants form small teams of three to four individuals to write manuscripts using the QI data. Manuscripts must be completed on the RAs’ personal time. Teams are responsible for initiating their own system of communication and style of working. Guidance is provided on authorship expectations and teams then determine authorship order based on interest and availability. Regular check-in meetings are held with each writing team to provide guidance and support. JP and GZ work closely with each team to review hypotheses and provide guidance on what statistical tests would be appropriate. They also review results with each team and provide guidance for interpreting the results. Some RAs express a particular interest in data cleaning and analysis; these RAs have the option to be more involved in the data cleaning and analysis process such as through reviewing analytic code or running analyses themselves with close guidance. Manuscripts extend beyond the 9-month didactic lecture series, thereby continuing the close collaboration across team members and mentors long term. Therefore, although the participants who have “completed” the program and are described below have finished the 9-month didactic series, the majority are still working with the authors on finishing their manuscripts. Thus, their involvement extends beyond 9 months for an undetermined amount of time. PREP participants are also encouraged to present posters at local, regional, or national conferences if interested.

The first QI project was focused on numerous issues related to increasing diversity and equity in our recruitment processes. The manuscripts that stemmed from this QI project include understanding facilitators to research participation and their association with demographic and pain characteristics, evaluating the role of mistrust of the medical system on one’s willingness to participate in pain-related research, and barriers to research participation and their association with demographic characteristics. Our second project aimed to better understand willingness to participate in clinical research interventions that are currently being studied at the B&PC. The manuscripts that stemmed from this QI project include examining predictors of willingness to participate in nonpharmaceutical pain interventions and the perception of graphical recruitment advertisements. Our current cohort is utilizing existing QI data that were collected by previous cohorts. They are currently working with the program directors (JP, SS, CSZ, and GZ) to identify QI-related research aims and hypotheses using these data.

## Participant feedback

Participants in the last two completed cohorts (2021–2022; 2022–2023) provided feedback on the program, specifically its impact on various domains of self-efficacy, job satisfaction, program satisfaction, and well-being. The feedback survey for the 2021–2022 cohort was distributed in March 2023, whereas for the 2022–2023 cohort it was distributed in April 2023. Some participants from both cohorts had ended their work with the team by that time, with many undergraduates finishing their semester of work or others moving away to medical school. Because the authors developed close relationships with the PREP participants, we opted to make the online survey anonymous and collect minimal demographic data to prevent our ability to identify the respondent; this was done with the hope of encouraging participation and honest responses. However, it also prevented the authors from reaching out to nonrespondents. The 2021–2022 cohort consisted of 12 participants, of which eight (67%) provided feedback. The 2022–2023 cohort consisted of six participants, of which four (67%) provided feedback.

Of the 12 participants who completed the feedback survey, 8 (67%) identified as female and 4 (33%) identified as male. At the beginning of their participation in PREP, most (*n* = 8/12; 67%) were employed by a research team at the University of Michigan; others were volunteer RAs or part of the UROP program. Most were undergraduate students (*n* = 4/12; 33%) or gap year students (*n* = 4/12; 33%), although three identified as medical students and one as a recent medical school graduate. Prior to beginning the program, a minority of participants saw research as part of their future career (*n* = 4/9; 44%). After participating in the program, a majority saw research as part of their future career (*n* = 9/10; 90%).

Overall, the participants rated the program positively. As seen in Table 2, most participants generally felt that the program helped them feel more involved in the research process, as well as more confident in their ability to develop and test a research question, develop a research poster, and prepare a manuscript. Many participants also reported that the program helped them feel a sense of belonging with their peers and more supported by peers and mentors. Many participants also reported that the program helped them feel more control over and invested in their typical work activities. Most participants felt that the program was valuable for their future and reported high satisfaction with the program. The QI project was rated as valuable by most participants, whereas the response to the lectures was more variable. Finally, the program appeared to have contributed positively to the well-being and job satisfaction of all participants, albeit to differing degrees.

**Table 2.** Participant feedback on PREP program (*n* = 12)

	Not at all	A little	Somewhat	Quite a bit	Very much
The PREP program helped me feel…					
More involved in the research process.			1 (8%)	3 (25%)	8 (67%)
More control over my day-to-day work activities.	1 (14%)		1 (14%)	1 (14%)	4 (57%)
More invested in my day-to-day work.	1 (11%)	1 (11%)	2 (22%)	1 (11%)	4 (44%)
More confident in my ability to develop and test a research question.			2 (17%)	6 (50%)	4 (33%)
More confident in my ability to develop a research poster.	1 (8%)		1 (8%)	4 (33%)	6 (50%)
More confident in my ability to write a manuscript.			1 (8%)	3 (25%)	8 (67%)
More confident in my ability to work effectively within a research team.		1 (8%)	1 (8%)	2 (17%)	8 (67%)
More confident in my ability to lead a team.		1 (8%)	4 (33%)	2 (17%)	5 (42%)
A sense of belonging among my peers.	1 (8%)		2 (17%)	4 (33%)	5 (42%)
More supported by my peers.	1 (8%)	1 (8%)	1 (8%)	3 (25%)	6 (50%)
More supported by mentors.				1 (8%)	11 (92%)
What I have learned in the PREP program will be valuable for my future.			1 (8%)	1 (8%)	10 (83%)
I was satisfied with the quality of my learning experience in the PREP program.				3 (25%)	9 (75%)
The lectures added value to my learning experience.		1 (8%)	4 (33%)	2 (17%)	5 (42%)
The QI project and manuscript writing added value to my learning experience.		1 (8%)		3 (25%)	8 (67%)
The PREP program improved my well-being.		3 (25%)	3 (25%)	3 (25%)	3 (25%)
The PREP program improved my job satisfaction.			3 (25%)	5 (42%)	4 (33%)

PREP = Pain Research Enrichment Program. QI = Quality Improvement.

Participants also provided qualitative feedback on the program. Table 3 displays the perceived positive impact of the program and what participants liked best about the program. For the positive impact of the program, most (*n* = 8/12; 67%) mentioned the value of learning more about the research process. A majority also mentioned the value of manuscript writing (*n* = 7/12; 58%) and half mentioned social support from peers and mentors (*n* = 6/12; 50%). Regarding what they liked best about the program, most participants mentioned the support from peers and mentors (*n* = 8/12; 67%), whereas a minority mentioned learning more about the research process (*n* = 4/12; 33%) and being involved in manuscript writing (*n* = 4/12; 33%).

**Table 3.** Participant qualitative feedback on PREP program: positive impact (*n* = 12)

**What positive impact did the program have on you or your work?**
Comfort in research, leading teams, writing papers for publication
I learned a lot about writing manuscripts which I found important to me as that is one of the essential functions of research.
The greatest positive impact [PREP] had was how much it taught me about manuscript writing and conducting research. The lectures, meetings with [mentors], and the process of writing the manuscript/making the poster were super valuable. I now feel much more comfortable with this process and am proud of the work we have done so far. I feel capable of doing a literature review and writing a manuscript, though I of course still need the help … with data analysis.
Skill building in the entire research process, giving me a community, providing me with outstanding mentors
It definitely made me enjoy research again. I used to work in a lab but hated it, I preferred [PREP]’s research process.
I am writing a manuscript for my job and PREP really helped me understand how this actually works.
Speech
It allowed me to feel more comfortable in research setting outside of recruitment.
Made me feel good since I didn’t think it would be possible to be involved in a manuscript and poster. Gave me a confidence boost and made me feel more involved in the team and projects!
I was introduced to the world of research and met some really amazing people who helped me understand the research process and get involved directly.
Having a well-rounded application is important for residency. Until I became a research assistant at UofM and subsequently joined [PREP] I had zero research experience and did not really know how to acquire any. [PREP] provided invaluable research opportunities including being a part of a manuscript publication and multiple poster presentations.
Learn more about the start and finish of clinical research. Get to answer research questions. Broaden my experience of research and work with peers with similar levels of experience and interest
**What did you like best about the PREP program?**
Getting to learn about scientific writing and then putting it into practice with my own research.
How to find and recognize mentors.
I liked the talk about how to write a manuscript and when we broke down literature review because no one had ever actually taught me how to do this.
Mentorship and manuscript writing/poster opportunities
QI projects
Working with everyone!
The mentors and the supportive environment.
The ability to interact with different people on the team in a different capacity than just to complete a job.
Structuring research from bare bones topics, great for people across all experience levels and gives a true understanding of what goes into the process. I really enjoyed when we all presented at the research symposium together and were all there to support each other including mentors and other study staff :)
The people, they were always so kind and helped me out whenever I needed help
[PREP] gave individuals with limited experience a gateway into the field of research. I felt well supported. For someone like me who had zero previous research experience, it was a nice first step into the realm of research.
Getting to start a project from scratch and go through all the different stages of the research including presenting our findings.

PREP = Pain Research Enrichment Program. QI = Quality Improvement.

Table 4 provides information on whether the program influenced future career decisions. Eleven of the 12 participants provided feedback. Overall, 45% (*n* = 5/11) indicated that the program influenced their career choices, 37% (*n* = 3/11) stated it did not, and 37% (*n* = 3/11) were unclear or uncertain. Of those who indicated that it did impact their career decisions, 60% (*n* = 3/5) suggested that it gave them direction or insight and 40% (*n* = 2/5) suggested that it inspired dedication to a career in pain management or pain research.

**Table 4.** Participant qualitative feedback on PREP program: future career (*n* = 11)

**Did PREP influence your future career decisions? If yes, how?**
I am not so sure it has influenced my career decision, I believe the experience will aid me in my journey to my final career of being a physician.
I think it confirmed that I can see myself doing research in my career.
It definitely taught me to be more comfortable with research and manuscript writing, which is a pretty important aspect of any medical career.
It made me think more about having research as a part of my career.
No, just helped me learn more about research.
Not explicitly, I enjoyed the research we did though.
Yes - inspired me to go into pain management.
Yes - my dedication to chronic pain broadly was increased significantly due to PREP.
Yes, it helped me get a direction on where I want to go next in my research career.
Yes, it showed multiple different aspects of research and what is involved in being the creative director behind these larger questions. Also gave me an insight to more data analysis and what is possible with different datasets you have.
Yes, I realized that I do enjoy scientific writing. I could imagine writing about medical research and contributing to my field, which excites me. I am much more open to the possibility of contributing to medical research in my career.

PREP = Pain Research Enrichment Program.

Seven of the 12 participants also provided feedback on the negative impact of the program. Two individuals mentioned feeling overwhelmed by the scope of the manuscript writing project and three mentioned that some of the lectures were not applicable to their interests or were not as engaging. One individual mentioned that they experienced some uncertainty about the timeline of the process and expressed poor communication within their writing team. One individual mentioned concerns related to time management. Nine of the 12 participants indicated what they liked least about the program. Three mentioned that some of the lecture topics were less enjoyable. Two individuals felt that the timeline and communication from the authors were slow. Two expressed the uncertainty of where they were in the process. Two mentioned that being entirely remote was not ideal and that they would appreciate seeing each other face to face. Finally, one individual indicated problems with communication within their project team.

Regarding deliverables, one manuscript has been accepted for publication [8], two manuscripts are currently under review in peer-reviewed journals, and four additional manuscripts are in progress. Six posters have been presented at local conferences, and two posters have been presented at regional conferences.

## Discussion

Overall, the PREP program has been successful in providing an enriched research experience for RAs. Participants generally expressed increased self-efficacy in various domains of the research process. They also reported that the PREP program contributed to an increased sense of social support from both peers and mentors. Indeed, social support from peers and mentors was reported to be the best part of the program by most of the participants. Most importantly, many reported the PREP program as contributing positively to their well-being and job satisfaction. Previous research has similarly shown the importance of building practical work-related skills and engaging in active learning experiences for building a sense of preparedness as young adults transition to the workforce [4]. Furthermore, previous research among medical residents suggest that mentoring was particularly important for perceived self-efficacy, whereas perceived social support contributed to positive affect and self-esteem [5].

Although the program was positive overall, participants’ negative experiences suggest areas for improvement. These improvements can be summarized by three primary domains: enhancing the QI experience; improving communication and support; and input on lecture topics.

### Enhancing the QI experience

Participants’ feedback suggests that the QI project and associated manuscript writing are the most valuable aspects of the program for their career development. Thus, future cohorts will maintain the QI project and manuscript writing and additional efforts will be made to augment this experience. Data collection for the QI projects has typically begun midway through the program. Our aim is to begin data collection earlier or utilize previously collected QI data in order to begin manuscript writing and mentorship sooner; this will increase the perceived applicability of the didactic lectures and also give us more time to grow mentoring relationships. Time will also be devoted during each didactic lecture meeting to discuss the timeline of the project and what to expect next. We will also hold brainstorming sessions with PREP participants on the applicability of their research findings to our processes at the B&PC.

### Improving communication and support

Some participants expressed that communication could be improved and experienced the project as overwhelming. Therefore, we will be holding more frequent, short “check-ins” with the writing teams to provide support, quick feedback, and advice. We also acknowledge the importance of discussing failure and self-doubt to destigmatize these very common academic experiences [6]. Thus, we plan to hold more formal and informal discussions on these topics to improve support. Although some participants indicated that they would like to meet in-person, we believe the fully remote design of the program maximized participation. Some RAs work for teams located in other places on campus or occasionally work from home. By meeting remotely, participants can participate regardless of their location. This is also ideal given that sessions are held during work hours and minimizing travel time ensures that the workday is not severely impacted.

### Input on lecture topics

Some participants expressed that the lecture topics were not applicable or engaging. Therefore, we plan to obtain feedback on the lectures in the beginning and end of the program and make adjustments to meet the participants’ needs.

There are numerous limitations that must be acknowledged. The goal of this descriptive paper was to simply provide an overview of a training program for RAs. We did not collect pre- and post-program data, nor did we have a control group with which to compare. Thus, this is not a systematic program evaluation. Feedback was collected at different lengths of time for each cohort, which may have led to differences in how those groups responded. Additionally, the sample size is very small and many participants did not respond to the feedback survey, potentially biasing our results. We prioritized anonymity to encourage honest responses, but this prevented us from reaching out to nonrespondents. Finally, this is a small program for which the authors serve as the mentors. We cannot account for differential mentoring effects that would likely impact similar programs in other research groups. However, despite these limitations for the interpretation of the current pilot program, we are hopeful that the description of the program and its potential benefits will be useful for other research teams that work with temporary RAs contemplating their future career and education.

## Conclusion

The PREP program in the Department of Anesthesiology at the University of Michigan has been successful in providing support and an enhanced research experience for temporary RAs. Integrating a combined didactic and practical training program with work-related duties positively impacted feelings of self-efficacy, job satisfaction, and well-being, as well as provided opportunities for career exploration.

## References

[1] American Psychological Association. Stress in America 2020: A National Mental Health Crisis. Washington, DC: American Psychological Association; 2020.

[2] Barbayannis G , Bandari M , Zheng X , Baquerizo H , Pecor KW , Ming X. Academic stress and mental well-being in college students: correlations, affected groups, and COVID-19. Front Psychol. 2022;13:886344. 35677139 10.3389/fpsyg.2022.886344 PMC9169886

[3] Deci E , Olafsen A , Ryan R. Self-determination theory in work organizations: the state of a science. Annu Rev Organ Psych. 2017;4(1):19–43.

[4] Garcia-Aracil A , Monteiro S , Almeida L. Students’ perceptions of their preparedness for transition to work after graduation. Act Learn High Educ. 2021;22(1):49–62.

[5] Giblin F , Lakey B. Integrating mentoring and social support within the context of stressful medical training. J Soc Clin Psychol. 2010;29(7):771–796.

[6] Jaremka LM , Ackerman JM , Gawronski B , et al. Common academic experiences no one talks about: repeated rejection, impostor syndrome, and burnout. Perspect Psychol Sci. 2020;15(3):519–543. 32316839 10.1177/1745691619898848

[7] Karyotaki E , Cuijpers P , Albor Y , et al. Sources of stress and their associations with mental disorders among college students: results of the world health organization world mental health surveys international college student initiative. Front Psychol. 2020;11:1759. 32849042 10.3389/fpsyg.2020.01759 PMC7406671

[8] Start C , McBride M , Zhu G , Shaikh S , Pierce J. Understanding facilitators of research participation among adults with self-reported chronic pain - a survey examining hypothetical research participation. BMC Med Res Methodol. 2024;24(1):18. 38254022 10.1186/s12874-023-02128-8 PMC10802039

